# Low Frequency Electrical Stimulation Either Prior to Or after Rapid Kindling Stimulation Inhibits the Kindling-Induced Epileptogenesis

**DOI:** 10.1155/2017/8623743

**Published:** 2017-03-08

**Authors:** Mostafa Jalilifar, Ali Yadollahpour, Ahmad Ali Moazedi, Zohreh Ghotbeddin

**Affiliations:** ^1^Department of Medical Physics, Faculty of Medicine, Ahvaz Jundishapur University of Medical Sciences, Ahvaz 6135715794, Iran; ^2^Department of Biology, Faculty of Science, Shahid Chamran University of Ahvaz, Ahvaz 6135743337, Iran; ^3^Department of Physiology, Faculty of Veterinary Medicine, Shahid Chamran University of Ahvaz, Ahvaz 6135743337, Iran

## Abstract

*Objective*. Studies are ongoing to find appropriate low frequency stimulation (LFS) protocol for treatment of epilepsy. The present study aimed at assessing the antiepileptogenesis effects of LFS with the same protocol applied either just before or immediately after kindling stimulations.* Method*. This experimental animal study was conducted on adult Wistar rats (200 ± 20 g) randomly divided into kindle (*n* = 7), LFS + Kindle (*n* = 6), and Kindle + LFS groups (*n* = 6). All animals underwent rapid kindling procedure and four packages of LFS (1 Hz) with 5 min interval were applied either immediately before (LFS-K) or after kindling stimulation (K-LFS). The after discharge duration (ADD), daily stages of kindling, and kindling seizure stage and number of stimulations required to reach each stage were compared between the three groups using two-way analysis of variance (ANOVA) followed by Tukey post hoc and one-way ANOVA, and Kruskal-Wallis test, respectively.* Results*. LFS in both protocols significantly decreased the ADD (*p* < 0.05) and daily seizure stages (*p* < 0.05) and increased the number of stimulations required to achieve stage 3 and stages 4 and 5 of kindling compared with the kindle group (stage 2: *p* > 0.05, stages 3 to 5: *p* < 0.05).* Conclusion*. Although LFS-K showed more inhibiting effect than K-LFS, the difference was not statistically significant.

## 1. Introduction

According to the recent World Health Organization (WHO) report, approximately 50 million people suffer from epilepsy worldwide and it is appraised that nearly 2.4 million new cases occur each year [1]. Temporal lobe epilepsy (TLE) is the most common type of epilepsy comprising more than 60% of all epileptic disorders [1, 2]. TLE consists of complex partial seizures originating from a small area of the temporal lobe or frontal lobe of the brain and quickly generalize to other areas of the brain [3].

Kindling is the most common experimental model of TLE used to study the development of human epilepsy [4–10]. It is a chronic technique where the consecutive electrical stimulation of specific areas of the brain increases seizure susceptibility and it also produces other changes in brain functions which are similar to those appearing in TLE patients [11–13]. Different kindling models have been employed to demonstrate the procedure for kindling-induced epileptogenesis but electrical kindling has gained much attention due to noninterference of chemical and pharmacological agents in the result of experiments. Electrical kindling was first described by Goddard et al. [14] and more research on the characteristics of the kindling revealed that it can be an acceptable option to simulate human epileptogenesis, learning, and memory. Later, Lothman et al. [15–17] introduced rapid kindling (RK) protocol that made animals fully kindled faster than other approaches.

It is evident that the efficacy of an epileptic treatment technique can be considerably improved by predicting seizure [18]. Given the unpredictable characteristic of less-controlled seizures, seizure prediction is a fundamental goal in clinical management [19]. In the majority of epileptic cases, Anti-Epileptic Drugs (AEDs) cannot completely eliminate epileptic seizures and they only reduce seizure severity [20]. Another traditional treatment is surgery that can only be used in patients with focal epilepsy and it may impair memory [21]. In addition, each of the current therapeutic options has its own side effects. In spite of developing various current traditional treatments such as pharmacological drugs and surgery, a large number of patients have experienced little improvement or suffered serious side effects. Therefore, finding a new and safe alternative therapeutic method is required. Vagus nerve stimulation (VNS) has been widely employed as an alternative for treatment of drug resistant epileptic patients [22, 23]. In this regard, Zhang et al. [24] reported that VNS decreases seizure frequency and it can be a safe and effective treatment for pharmacoresistant epileptic patients. Recently, application of electrical stimulation, especially low frequency stimulation (LFS), of brain as an antiepileptic method has gained considerable attention [25]. Administration of LFS is considered to inhibit the seizure activity by inducing long term depression (LTD) and removing kindling-induced synaptic potentiation [26]. The antiepileptogenic role of LFS during the kindling procedure was first introduced by Gaito et al. [27, 28]. Following that, several studies revealed that LFS can prevent generalization of seizures and inhibit boosting induction of the kindling phenomenon through alteration of electrophysiological features and inhibition of the synaptic activity [20, 25, 29–32]. In addition, electrical stimulation provides various privileges in comparison with other current therapies. It is more secure and, up to now, its serious side effects have not been reported [33–35]. Thus, LFS is considered as an alternative to the brain surgery for patients with epilepsy because of its low risk and being less invasive [36].

Several studies have demonstrated the inhibiting effects of LFS during the kindling process, highlighting the idea that LFS can be regarded as a good alternative therapy for epileptic patients [20, 29, 37]. In order to develop this technique, few studies have investigated the ability of LFS applied immediately before epileptic discharges or kindling stimulation to prevent the seizure attacks and most of them applied LFS some minutes before kindling stimulation [20, 38–41]. On the other hand, developing responsiveness closed loop systems for prediction and prevention or early treatment of seizures has drawn a considerable amount of research interest. One of the main prerequisites of such systems is an efficient therapeutic agent capable of being applied just before epileptic seizures to prevent or inhibit them [42]. LFS seems to meet this criterion if applied in optimum protocols and time. Developing such effective LFS protocol can be used in responsiveness closed loop system for predicting and preventing seizure attacks. The present study aimed to comparatively assess the antiepileptogenic effects of 1 Hz LFS with the same protocol applied just before and immediately after the kindling.

## 2. Materials and Methods

### 2.1. Animals

Adult male Wistar rats weighing 200 ± 20 g were obtained from the animal house of Ahvaz Jundishapur University of Medical Sciences (AJUMS), Ahvaz, Iran. They were maintained in a colony room with a constant temperature (23 ± 2°C), humidity (50 ± 5%), and artificial 12 : 12 h light-dark cycle. The lights were turned on at 7:00 AM. Animals were housed in individual cages with woodchip bedding and had free access to standard food and water. Efforts were made to reduce animal suffering and to minimize the number of used animals. All the experimental procedures of this study were performed in accordance with the ethical guidelines set by the local Ethical Committee of AJUMS (Reg. code; U-94147) which completely coincide with the “NIH Guide for the Care and Use of Laboratory Animals.” All experiments were done within the same day time (9:00 AM to 5:00 PM). The experiments lasted for 8 months from 14 September 2015 to 28 May 2016.

### 2.2. Surgery

The animals were anesthetized using a mixture of ketamine (100 mg/kg, i.p.) and xylazine (20 mg/kg, i.p.) [20]. The anesthetized rats were fixed in a stereotaxic instrument. Three holes were drilled including one for an anchor screw, one for placement of a monopolar stainless steel electrode used as ground and reference electrode in the front of skull, and another hole for implantation of tripolar stainless steel electrode (A-M system, USA) (a bipolar for stimulating and a monopolar for recording EEG signals) that was implanted in the right amygdale, using Paxinos and Watson atlas coordinates from Bregma: anteroposterior: −2.5 mm; lateral: 4.8 mm; vertical: 7.2 and 0.2 mm below the skull [43]. Electrodes were embedded with acrylic dental cement and attached to a socket.

### 2.3. Stimulation Procedures

**Figure 1:**
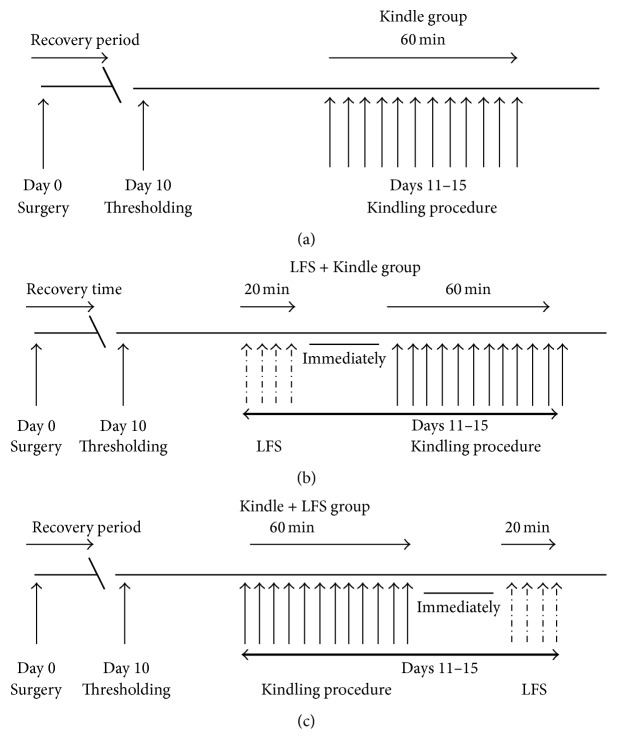
Schematic diagram of the study design and experiments in Kindle group (a), low frequency stimulation (LFS) + Kindle group (b), and Kindle + LFS group (c).

Ten days after surgery, the threshold intensity of kindling stimulation protocol, after discharge (AD) threshold, was determined. The AD threshold was determined by 1 ms monophasic square-wave of 50 Hz with 3 s train duration as described previously [43]. The stimulating currents were initially delivered at 10 *μ*A and then its intensity was increased in increments of 10 *μ*A at 5 min intervals. The minimum intensity sufficient to induce the ADs for at least 8 s was selected as the AD threshold and used for kindling stimulation. The AD was defined as spikes with a frequency of at least 1 Hz and amplitude of at least twice the baseline activity originating immediately after stimulation [43, 44]. Electrical stimulations were applied using an electromodulator device (Sciencebeam Co., Tehran, Iran) which was connected to a monitor to show EEG signal alterations using the e-probe software. The rats which elicited no AD with a current intensity of up to 350 *μ*A were removed from the experiment. Animals were randomly divided into 3 groups: the Kindle group (*n* = 7), the LFS + Kindle group (LFS-K) (*n* = 6) where LFS was applied just before the kindling stimulations, and Kindle + LFS group (K-LFS) (*n* = 6) where LFS was administered immediately after the kindling stimulations. All animals were subjected to a rapid kindling stimulation (a 3 s train of 50 Hz monophasic pulses of 1 ms duration with the threshold intensity applied 12 times daily). The kindling progression was scored according to the Racine scale: stage 1: facial clonus, wet dog shakes, and mouth; stage 2: facial movement and head nodding; stage 3: forelimb clonus; stage 4: rearing and tonic extension of forelimbs; stage 5: falling and loss of balance [13]. The kindling stimulations continued until emergence of stage 5 of kindling (it took 4.42 days). Therefore, the LFS-K and K-LFS animals were stimulated for 5 days. Four packages of LFS (each package consisted of 200 monophasic square pulses, 0.1 ms pulse duration at 1 Hz with the AD threshold intensity) with 5 min interval were daily applied immediately either before the kindling stimulations (LFS-K group) or after the termination of the kindling stimulations (K-LFS group). The time between the LFS and kindling stimulation was as short as technical limitation allowed. However, the time did not exceed more than 1 min. The schematic diagram of the experimental groups is presented in Figure 1.

### 2.4. Statistical Analyses

Values were averaged and expressed as the mean ± Standard Error of Mean (SEM). A two-way analysis of variance (ANOVA) was performed to compare the changes in the values of after discharge duration (ADD). Moreover, the significant differences were determined using a post hoc of Tukey. A one-way* analysis* of* variance* (ANOVA) was employed to determine differences in ADD between three groups during each day. The behavioral stages of kindling and the number of stimulations required to achieve different seizure stages were compared using Kruskal-Wallis followed by Bonferroni's test. Statistical analysis was done by statistical package of SPSS (*version 21*, IBM Corporation, Chicago, IL, USA) for windows. For all statistical analyses, the tests were carried out as two-sided and significance was set at *p* < 0.05.

## 3. Results

### 3.1. Alterations in after Discharge Duration

**Figure 2:**
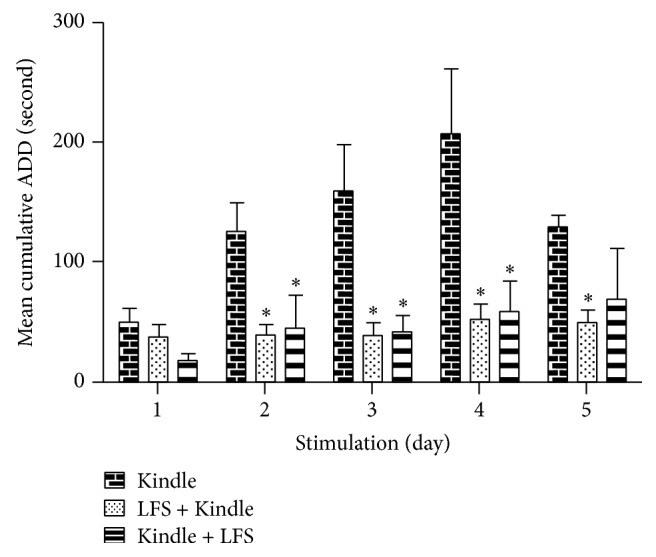
Comparison of after discharge duration (ADD) in 5 days of stimulation between 3 groups (Kindle, LFS + Kindle, and Kindle + LFS). LFS either prior to or after kindling significantly reduced ADD compared with Kindle group. In addition, the Kindle + LFS and LFS + Kindle groups showed no significant difference (*p* > 0.05). Values are represented as mean ± Standard Error of Mean (SEM). ^*∗*^*p* < 0.05 compared with Kindle group. LFS: low frequency stimulation.

A two-way ANOVA showed significant difference in cumulative ADD between the experimental groups (*F*[2,78] = 19.682, *p* < 0.05). The data demonstrated that the cumulative ADD decreased significantly as a result of applying LFS in both LFS-K and K-LFS groups as compared with Kindle group (*p* < 0.05). Although ADD in LFS-K group was lower than K-LFS, the difference was not statistically significant (*p* > 0.05). Along with the kindling acquisition, the difference between Kindle group and LFS-K and K-LFS groups increased (*p* > 0.05) (Figure 2).

### 3.2. Seizure Stage

**Figure 3:**
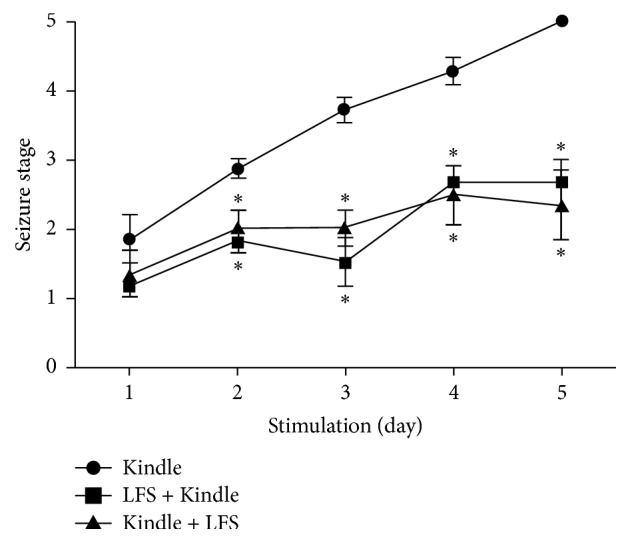
Comparison of behavioral seizure stages during 5 days of stimulation between 3 groups. LFS significantly inhibited seizure progression. A Kruskal-Wallis followed by Bonferroni's test showed significant decrease of seizure stage in both Kindle + LFS and LFS + Kindle groups compared with Kindle group (*p* < 0.05). The Kindle + LFS and LFS + Kindle groups showed no significant difference (*p* > 0.05). Data are shown as mean ± Standard Error of Mean (SEM). *∗* denotes *p* < 0.05 compared with Kindle group. LFS: low frequency stimulation.

The results showed no significant difference in daily stage between all groups during the first day of the experiment (*p* > 0.05). However, application of LFS at the 2nd, 3rd, 4th, and 5th days significantly suppressed the daily stages (for day 2:*** H*[**2**]** = 10.191, *p* < 0.05, for day 3:* H*[2] = 13.696, *p* < 0.05, for day 4:* H*[2] = 12.003, *p* < 0.05, and for day 5:* H*[2] = 10.667, *p* < 0.05). All animals in the Kindle group achieved the generalized seizure stages (stages 4-5 of kindling) at most at the 5th day. Administration of LFS could significantly prevent the behavioral progression of seizure as at the end of the experiment only one animal in LFS-K group (16%) and two animals (32%) in K-LFS groups reached generalized seizure stage.  Figure 3 compared the daily stage of animals during 5 consecutive days' stimulation between 3 groups. Applying the LFS either before or after kindling stimulation significantly inhibited the epileptogenesis (*p* < 0.05). Though the LFS-K showed more inhibition effect as compared with the K-LFS protocol, the difference was not statistically significant (*p* > 0.05).

### 3.3. Stimulation Number

**Figure 4:**
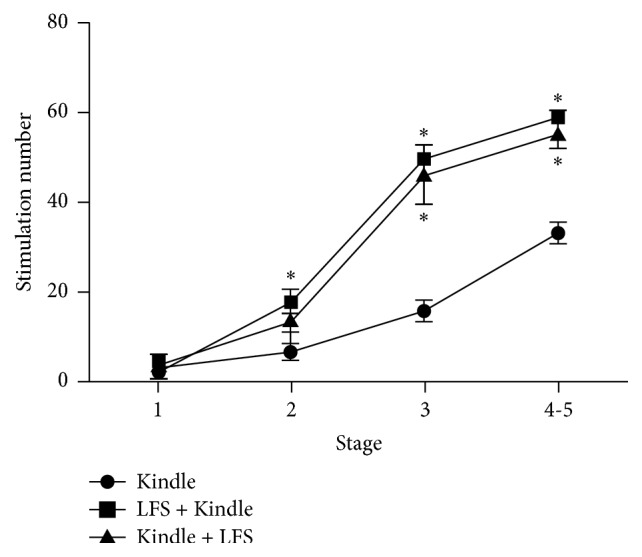
Effect of LFS on stimulation numbers required to achieve each seizure stage. Both Kindle + LFS and LFS + Kindle groups showed significant increase in stimulation numbers required to reach kindling stages compared to Kindle group (*p* < 0.05). Although LFS + Kindle group required more stimulation numbers to achieve localized (stage 3) and generalized seizure stages (stages 4-5), compared to Kindle + LFS rats, the difference was not statistically significant (*p* > 0.05). The values are represented as mean ± SEM. *∗* denotes *p* < 0.05 compared with Kindle group. LFS: low frequency stimulation.

A Kruskal-Wallis followed by a Bonferroni's test showed that applying LFS either before or after kindling stimulations significantly increased the number of stimulations required to achieve localized (stage 3) and generalized (stages 4 and 5) seizure stages (for stage 2:* H*[2] = 6.725, *p* > 0.05, for stage 3:* H*[2] = 8.498, *p* < 0.05, and for stages 4-5:* H*[2] = 13.658, *p* < 0.05). All animals of Kindle group showed the seizure generalization within an average of (32.86 ± 1.97) trials, whereas at the end of 60 trials, only one animal of LFS-K and two of K-LFS group showed seizure generalized stage (Figure 4). In this assessment, LFS-K showed stronger inhibiting effect than K-LFS, whereas the difference was not significant. To reach stage 3 and stages 4-5, the LFS-K, respectively, received 49.83 ± 8.03 and 59.0 ± 2.04 stimulations, whereas K-LFS received 46.16 ± 16.50 and 55.33 ± 7.89, respectively.

## 4. Discussion

The results confirmed that applying LFS either before or after the kindling stimulation significantly inhibited the kindling acquisition procedure. All LFS-K and K-LFS animals showed lower ADD and daily stages in comparison with the Kindle group. LFS could also considerably prevent generalization of behavioral stages during the kindling procedure. Moreover, all animals treated by LFS required more stimulations to show generalized seizure stages. The findings correspond with most of the previous studies which confirmed the inhibition effect of LFS during the kindling acquisition [25, 30, 37]. In addition, applying LFS reduced ADD which can be attributed to the retarding seizure generalization.

Due to the flexibility of LFS protocols, numerous studies have investigated different parameters involved in using this approach [20, 44, 45]. For example, Shahpari et al. [44] reported that the frequency of LFS and the interval time between LFS and the kindling stimulation play an important role in the extent of effectiveness of LFS. They reported that 4 packages of LFS showed the stronger antiepileptic effect than 1 and 8 packages. In addition, they found that applying LFS at 0.25 Hz provides more inhibitory effect than other frequencies. However, there was no association between the number of pulses and the amount of the antiepileptic effect of LFS [44]. Therefore, it seems that various LFS parameters can affect its anticonvulsant effect. Although the inhibitory effect of LFS has been widely reported, its exact antiepileptogenesis mechanism still remains unknown. The mechanism of long term depression (LTD) has been demonstrated to interfere with increasing the threshold of ADD and suppressing the generalization of seizures [20, 45–47]. In this regard, several studies have revealed that LFS-induced LTD [29, 31] and low level direct current stimulation-induced LTD [48] during the kindling acquisition can prevent the progression of seizure. The LTD phenomenon was first introduced in the hippocampus and then it was recognized in the other areas of the brain [49]. This effect can also appear in different neurons which release neurotransmitters. The most common neurotransmitter involved in inducing LTD is glutamate which affects some receptors causing inhibition effects on the synaptic activities. It was reported that the anticonvulsant effect of LFS may occur through inhibiting the synaptic transition in the dentate gyrus [50]. Similarly, another study examined the antiepileptogenic effect of LFS during the kindling acquisition of perforant path and they suggested that the inhibiting effect of LFS is caused by the suppression of synaptic transmission in dentate gyrus. They also found that LFS prevents the kindling-induced enhancement of pulses depotentiation [51]. In addition, a large amount of ATP and adenosine products were released from the end of presynaptic to synaptic regions during exciting the CA1 region of the hippocampus in the kindling process. In this regard, administration of LFS enhances the release of adenosine which in turn induces LTD in neurons of the CA1 region [52]. Some studies have reported that adenosine induces the inhibitory effect through affecting A1 receptors and the expression of these receptors was increased as a result of applying LFS [50, 53]. In addition, it was showed that the application of the A1 receptors agonist during LFS increases LTD phenomenon [52]. Due to the role of the A1 receptors in the inhibiting effect of LFS, enhancement of the extracellular amount of adenosine can increase the antiepileptic effect of LFS. Therefore, applying LFS induces alteration in the adenosine receptors which may be associated with the increase of the AD threshold and reduction of seizure susceptibility during the kindling procedure [54]. Similar to the results, numerous studies have revealed LFS-induced reduction of ADD during the kindling process [20, 32, 44], whereas Toibaro et al. [21] reported that the reduction was not statistically significant. However, comparing the findings of our study with other previous similar studies it is important to consider the difference between the LFS parameters particularly the exact time of LFS application. In our study, we applied the LFS stimulation immediately before or after kindling stimulation, whereas in most of the other similar studies there was an interval between LFS and kindling of mostly 5 minutes. Esmaeilpour et al. (2013) used 8 packages at 100 s interval, each package consisting of 200 monophasic square-wave pulses, 0.1 ms pulse duration at 1 Hz [20], and Wu et al. (2013) applied LFS twice per day for two weeks [32]. Shahpari et al. (2012) used several protocols of LFS at 1, 0.25, and 5 Hz [44]. They also applied the protocol used in this experiment but the protocol was applied in the perforant path region whereas we stimulated the amygdala.

In the present study, application of LFS either before or after the kindling stimulation significantly decreased ADD, compared with the Kindle group; however, the difference between the K-LFS and LFS-K groups was not statistically significant. It seems that applying LFS either immediately before or after kindling stimulation has the same antiepileptogenesis effects.

The findings showed that LFS can suppress behavioral seizure generalization which agreed with the earlier studies [20, 21, 55–57]. All animals in the Kindle group experienced generalized seizure stages within 5 days, whereas only one animal in the LFS-K group and two animals in the K-LFS group achieved stages 4 and 5 of the kindling. Moreover, application of LFS in both LFS-K and K-LFS protocols significantly increased the number of stimulations required to achieve generalized seizure stages. In fact, this inhibition effect might be due to the delay of the behavioral progression from stages 0–3 to stages 4-5. It is clear that increasing the seizure threshold would reduce the risk of behavioral seizure generalization. Thus, application of LFS following the kindling procedure can increase the seizure threshold through the LTD process which retards network synchronization and suppresses seizure progression. This can justify requiring more stimulations in the LFS-K and K-LFS groups as compared with the Kindle group animals to demonstrate each seizure stage [21].

To sum up, applications of the same LFS protocol just before or immediately after the kindling stimulations significantly inhibit the kindling-induced epileptogenesis and the LFS before the kindling stimulations exerts higher inhibiting effects than the later protocol but the difference was not significant. In a similar study, Shahpari et al. [44] compared the antiepileptic effect of the same LFS protocol applied immediately before kindling stimulation and 5 min after termination of kindling stimulation and reported that LFS protocol immediately before the kindling stimulation induced more inhibiting effects [44]. However, the main difference between this study and that of Shahpari et al. [44] is that we applied LFS immediately after kindling termination which can be attributed to the different antiepileptic effect between immediately after LFS and LFS applied 5 min after stimulation. Five minutes is a long enough time to propagate epileptic activities through other regions of brain particularly in stages 3–5; therefore, applying LFS immediately after kindling stimulation more likely inhibits the epileptic activities. However, in addition to the time variable of applying LFS, there are many other factors that should be discussed and examined in order to use LFS as a therapeutic approach. During the recent years, a considerable amount of research interest has been devoted to developing seizure predicting approaches for efficient management of epileptic disorders [19]. Developing an open loop or closed loop system capable of predicting seizure attack or early detection of seizure onset and applying efficient electrical stimulations to prevent or at least impede the seizure attacks is one of the management options for patients with intractable epilepsy. In this regard, finding appropriate parameters of electrical stimulations particularly time of application is a crucial step. The finding showed that the LFS can be used in such systems as its application either prior to or after the kindling stimulation, resembling seizure onsets, can significantly inhibit epileptogenesis.

The main limitation of this study was lack of cellular and molecular assessments to investigate the mechanisms of action of the two protocols of LFS. In addition, the time of LFS applications prior to or after kindling stimulation varies and was not exactly the same. This variation was because of the technical limitation in changing the stimulus paradigm from kindling to LFS and vice versa. However, we tried to apply all LFS prior to or after kindling with minimum variation and all the stimulations were performed within 1 min interval with kindling stimulation. In addition, possible interfering effects of surgery on the study parameters were another source of the limitation of this study. However, we have a kindling group and comparing the parameters between the three groups we can expect that any possible effects of the surgery could be similar in all animals.

## 5. Conclusion

The findings showed that LFS (four packages, each package consisting of 200 monophasic square pulses, 0.1 ms pulse duration at 1 Hz) applied immediately before or after kindling stimulations can significantly inhibit kindling-induced epileptogenesis. It seems that LFS or even high frequencies of electrical stimulation immediately or in close interval before kindling stimulations can be used as an efficient technique in closed loop seizure prediction and prevention system to prevent or inhibit epileptic discharges. Performing further studies to find more efficient protocols capable of damping epileptic discharges is necessary in this regard.

## References

[1] McCandless D. W. (2012). *Epilepsy: Animal and Human Correlations*.

[2] Sloviter R. S. (2005). The neurobiology of temporal lobe epilepsy: too much information, not enough knowledge. *Comptes Rendus Biologies*.

[3] Sen A. K., Kubek M. J., Shannon H. E. (2007). Analysis of seizure EEG in kindled epileptic rats. *Computational and Mathematical Methods in Medicine*.

[4] Schwartzkroin P., Engel J., Pitkänen A., Schwartzkroin P., Moshé S. (2006). What good are animal models. *Models of Seizures and Epilepsy*.

[5] Jefferys J. G. (2003). Models and mechanisms of experimental epilepsies. *Epilepsia*.

[6] Coulter D. A., McIntyre D. C., Löscher W. (2002). Animal models of limbic epilepsies: what can they tell us?. *Brain Pathology*.

[7] Fisher R. S. (1989). Animal models of the epilepsies. *Brain Research Reviews*.

[8] Theodore W. H., Fisher R. S. (2004). Brain stimulation for epilepsy. *The Lancet Neurology*.

[9] Cole A. J., Koh S., Zheng Y. (2002). Are seizures harmful: what can we learn from animal models?. *Progress in Brain Research*.

[10] Bertram E. (2007). The relevance of kindling for human epilepsy. *Epilepsia*.

[11] Sato M., Racine R. J., McIntyre D. C. (1990). Kindling: basic mechanisms and clinical validity. *Electroencephalography and Clinical Neurophysiology*.

[12] Racine R. J. (1972). Modification of seizure activity by electrical stimulation: I. after-discharge threshold. *Electroencephalography and Clinical Neurophysiology*.

[13] Racine R. J. (1972). Modification of seizure activity by electrical stimulation: II. Motor seizure. *Electroencephalography and Clinical Neurophysiology*.

[14] Goddard G. V., McIntyre D. C., Leech C. K. (1969). A permanent change in brain function resulting from daily electrical stimulation. *Experimental Neurology*.

[15] Lothman E. W., Salerno R. A., Perlin J. B., Kaiser D. L. (1988). Screening and characterization of antiepileptic drugs with rapidly recurring hippocampal seizures in rats. *Epilepsy Research*.

[16] Lothman E. W., Williamson J. M. (1993). Rapid kindling with recurrent hippocampal seizures. *Epilepsy Research*.

[17] Lothman E. W., Williamson J. M. (1994). Closely spaced recurrent hippocampal seizures elicit two types of heightened epileptogenesis: a rapidly developing, transient kindling and a slowly developing, enduring kindling. *Brain Research*.

[18] Iasemidis L. D. (2011). Seizure prediction and its applications. *Neurosurgery Clinics of North America*.

[19] Yadollahpour A., Jalilifar M. (2014). Seizure prediction methods: a review of the current predicting techniques. *Biomedical and Pharmacology Journal*.

[20] Esmaeilpour K., Masoumi-Ardakani Y., Sheibani V., Shojaei A., Harandi S., Mirnajafi-Zadeh J. (2013). Comparing the anticonvulsant effects of low frequency stimulation of different brain sites on the amygdala kindling acquisition in rats. *Basic and Clinical Neuroscience*.

[21] Toibaro L., Pereyra M., Pastorino J. (2012). Effect of unilateral low-frequency stimulation of hippocampus on rapid kindling—induced seizure development in rats. *Neuroscience & Medicine*.

[22] Handforth A., DeGiorgio C. M., Schachter S. C. (1998). Vagus nerve stimulation therapy for partial-onset seizures: a randomized active-control trial. *Neurology*.

[23] Bao M., Zhou J., Luan G.-M. (2011). Treatment of drug-resistant epilepsy with vagus nerve stimulation—review of 45 cases. *Chinese Medical Journal*.

[24] Zhang J., Meng F., Jia F. (2015). Vagus nerve stimulation for pediatric and adult patients with pharmaco-resistant epilepsy. *Chinese Medical Journal*.

[25] D'Arcangelo G., Panuccio G., Tancredi V., Avoli M. (2005). Repetitive low-frequency stimulation reduces epileptiform synchronization in limbic neuronal networks. *Neurobiology of Disease*.

[26] Weiss S. R. B., Li X.-L., Rosen J. B., Li H., Heynen T., Post R. M. (1995). Quenching: inhibition of development and expression of amygdala kindled seizures with low frequency stimulation. *NeuroReport*.

[27] Gaito J. (1980). The effect of variable duration one hertz interference on kindling. *The Canadian Journal of Neurological Sciences*.

[28] Gaito J., Nobrega J. N., Gaito S. T. (1980). Interference Effect of 3 Hz Brain Stimulation on Kindling Behavior Induced by 60 Hz Stimulation. *Epilepsia*.

[29] Goodman J. H., Berger R. E., Tcheng T. K. (2005). Preemptive low-frequency stimulation decreases the incidence of amygdala-kindled seizures. *Epilepsia*.

[30] Koubeissi M. Z., Kahriman E., Syed T. U., Miller J., Durand D. M. (2013). Low-frequency electrical stimulation of a fiber tract in temporal lobe epilepsy. *Annals of Neurology*.

[31] Velíšek L., Velíšková J., Stanton P. K. (2002). Low-frequency stimulation of the kindling focus delays basolateral amygdala kindling in immature rats. *Neuroscience Letters*.

[32] Wu G., Hong Z., Li Y., Zhou F., Shi J. (2013). Effects of low-frequency hippocampal stimulation on gamma-amino butyric acid type B receptor expression in pharmacoresistant amygdaloid kindling epileptic rats. *Neuromodulation*.

[33] Boon P., Vonck K., De Herdt V. (2007). Deep brain stimulation in patients with refractory temporal lobe epilepsy. *Epilepsia*.

[34] Nitsche M. A., Paulus W. (2009). Noninvasive brain stimulation protocols in the treatment of epilepsy: current state and perspectives. *Neurotherapeutics*.

[35] Kile K. B., Tian N., Durand D. M. (2010). Low frequency stimulation decreases seizure activity in a mutation model of epilepsy. *Epilepsia*.

[36] Li Y., Mogul D. J. (2007). Electrical control of epileptic seizures. *Journal of Clinical Neurophysiology*.

[37] Ghotbedin Z., Janahmadi M., Mirnajafi-Zadeh J., Behzadi G., Semnanian S. (2013). Electrical low frequency stimulation of the kindling site preserves the electrophysiological properties of the rat hippocampal CA1 pyramidal neurons from the destructive effects of amygdala kindling: the basis for a possible promising epilepsy therapy. *Brain Stimulation*.

[38] Chen Y.-L., Huang C.-C., Hsu K.-S. (2001). Time-dependent reversal of long-term potentiation by low-frequency stimulation at the hippocampal mossy fiber–CA3 synapses. *The Journal of Neuroscience*.

[39] Liu Y., Wang Y., Xu Z.-H., Chen Z. (2015). Antiepileptic effect of low frequency stimulation in kindling rats. *Zhejiang da xue xue bao. Yi xue ban: Journal of Zhejiang University. Medical sciences*.

[40] Rohani R., Piryaei A., Jahanshahi A., Sadeghi Y., Mirnajafi-Zadeh J. (2014). Effect of low-frequency stimulation on kindling induced changes in rat dentate gyrus: an ultrastructural study. *Acta Neurologica Belgica*.

[41] Ghorbani Moghadam P., Mohammad-Zadeh M., Mirnajafi-Zadeh J., Fathollahi Y. (2006). The effect of parameters of low-frequency electrical stimulation on piriform-cortex kindled seizures in rat. *Physiology and Pharmacology*.

[42] Ramgopal S., Thome-Souza S., Jackson M. (2014). Seizure detection, seizure prediction, and closed-loop warning systems in epilepsy. *Epilepsy and Behavior*.

[43] Yadollahpour A., Firouzabadi S. M., Shahpari M., Mirnajafi-Zadeh J. (2014). Repetitive transcranial magnetic stimulation decreases the kindling induced synaptic potentiation: effects of frequency and coil shape. *Epilepsy Research*.

[44] Shahpari M., Mirnajafi-Zadeh J., Firoozabadi S. M. P., Yadollahpour A. (2012). Effect of low-frequency electrical stimulation parameters on its anticonvulsant action during rapid perforant path kindling in rat. *Epilepsy Research*.

[45] Albensi B. C., Ata G., Schmidt E., Waterman J. D., Janigro D. (2004). Activation of long-term synaptic plasticity causes suppression of epileptiform activity in rat hippocampal slices. *Brain Research*.

[46] Cheong M. Y., Yun S. H., Mook-Jung I., Kang Y., Jung M. W. (2002). Induction of homosynaptic long-term depression in entorhinal cortex. *Brain Research*.

[47] Kemp N., Bashir Z. I. (2001). Long-term depression: a cascade of induction and expression mechanisms. *Progress in Neurobiology*.

[48] Weiss S. R. B., Eidsath A., Li X.-L., Heynen T., Post R. M. (1998). Quenching revisited: low level direct current inhibits amygdala-kindled seizures. *Experimental Neurology*.

[49] Massey P. V., Bashir Z. I. (2007). Long-term depression: multiple forms and implications for brain function. *Trends in Neurosciences*.

[50] Boison D. (2006). Adenosine kinase, epilepsy and stroke: mechanisms and therapies. *Trends in Pharmacological Sciences*.

[51] Mohammad-Zadeh M., Mirnajafi-Zadeh J., Fathollahi Y. (2007). Effect of low frequency stimulation of perforant path on kindling rate and synaptic transmission in the dentate gyrus during kindling acquisition in rats. *Epilepsy Research*.

[52] Fujii S., Kuroda Y., Ito K.-I., Kaneko K., Kato H. (1999). Effects of adenosine receptors on the synaptic and EPSP-spike components of long-term potentiation and depotentiation in the guinea-pig hippocampus. *Journal of Physiology*.

[53] Boison D. (2005). Adenosine and epilepsy: from therapeutic rationale to new therapeutic strategies. *The Neuroscientist*.

[54] Ackermann R. F., Finch D. M., Babb T. L., Engel J. (1984). Increased glucose metabolism during long-duration recurrent inhibition of hippocampal pyramidal cells. *Journal of Neuroscience*.

[55] Ghorbani P., Mohammad-Zadeh M., Mirnajafi-Zadeh J., Fathollahi Y. (2007). Effect of different patterns of low-frequency stimulation on piriform cortex kindled seizures. *Neuroscience Letters*.

[56] Yang L.-X., Jin C.-L., Zhu-Ge Z.-B. (2006). Unilateral low-frequency stimulation of central piriform cortex delays seizure development induced by amygdaloid kindling in rats. *Neuroscience*.

[57] Zhu-Ge Z.-B., Zhu Y.-Y., Wu D.-C. (2007). Unilateral low-frequency stimulation of central piriform cortex inhibits amygdaloid-kindled seizures in Sprague-Dawley rats. *Neuroscience*.

